# Antenatal screening for Group B Streptococcus: A diagnostic cohort study

**DOI:** 10.1186/1471-2393-5-12

**Published:** 2005-07-22

**Authors:** Janet E Hiller, Helen M McDonald, Philip Darbyshire, Caroline A Crowther

**Affiliations:** 1 Department of Public Health, University of Adelaide, Adelaide, Australia 5005; 2 Emeritus Microbiologist, Microbiology & Infectious Diseases Department, Women's & Children's Hospital, 72 King William Road, North Adelaide, South Australia, Australia 5006; 3 Department of Nursing & Midwifery Research & Practice Development, 2^nd ^floor, Samuel Way Building, Women's & Children's Hospital, 72 King William Road, North Adelaide, South Australia, Australia 5006; 4 Department of Obstetrics and Gynaecology, University of Adelaide, Adelaide, Australia 5005

## Abstract

**Background:**

A range of strategies have been adopted to prevent early onset Group B Streptococcal (EOGBS) sepsis, as a consequence of Group B Streptococcal (GBS) vertically acquired infection. This study was designed to provide a scientific basis for optimum timing and method of GBS screening in an Australian setting, to determine whether screening for GBS infection at 35–37 weeks gestation has better predictive values for colonisation at birth than screening at 31–33 weeks, to examine the test characteristics of a risk factor strategy and to determine the test characteristics of low vaginal swabs alone compared with a combination of perianal plus low vaginal swabs per colonisation during labour.

**Methods:**

Consented women received vaginal and perianal swabs at 31–33 weeks gestation, 35–38 weeks gestation and during labour. Swabs were cultured on layered horse blood agar and inoculated into selective broth prior to analysis. Test characteristics were calculated with exact confidence intervals for a high risk strategy and for antenatal screening at 31–33 and 35–37 weeks gestation for vaginal cultures alone, perianal cultures alone and combined low vaginal and perianal cultures.

**Results:**

The high risk strategy was not informative in predicting GBS status during labour. There is an unequivocal benefit for the identification of women colonised with GBS during labour associated with delaying screening until 36 weeks however the results for method of screening were less definitive with no clear advantage in using a combined low vaginal and perianal swabbing regimen over the use of a low vaginal swab alone.

**Conclusion:**

This study can contribute to the development of prevention strategies in that it provides clear evidence for optimal timing of swabs. The addition of a perianal swab does not confer clear benefit. The quantification of advantages and disadvantages provided in this study will facilitate communication with clinicians and pregnant women alike.

## Background

Group B Streptococcus (GBS) infection in infants as a consequence of vertically acquired infection, is an important cause of neonatal mortality and morbidity, presenting as sepsis or pneumonia [1]. The incidence of early onset group B streptococcus sepsis (EOGBS) occurring within the first week of life has fallen in Australia from 2.0 per 1000 live births in 1991–1993 to 0.5 per 1000 live births in 1995–1997 [2]. This figure is similar to the recently reported annual incidence of 0.48 per 1000 from the United Kingdom and Ireland [3].

Vaginal colonisation occurs in 11–30% of all pregnant women [4-6] and 50–75% of their infants become colonised usually during labour or birth. There is clear evidence that intrapartum colonisation is strongly associated with EOGBS sepsis [7] which has a case-fatality of approximately 4%[1]. Serious morbidities include sepsis, pneumonia, meningitis, osteomyelitis or septic arthritis.

The United States' Centers for Disease Control has endorsed a strategy in which screening of pregnant women is to occur at 35–37 weeks gestation using vaginal and rectal swabs and all women delivering before 37 weeks are to be treated if they are of GBS culture positive or of unknown GBS status, a change from their previous policy in which a strategy of intrapartum chemoprophylaxis based on a risk-based approach also was endorsed [8]. This contrasts with the 2003 recommendation from the Royal College of Obstetricians and Gynaecologists which states that "routine screening (either bacteriological or risk based) for antenatal GBS carriage is not recommended" [9]. There is no standard accepted approach to the prevention of EOGBS. Strategies have evolved including screening antenatally to detect colonisation or treatment of women with risk factors including prolonged rupture of membranes, intrapartum fever, preterm labour and history of maternal colonisation during pregnancy reflecting in part, the impact of local data on the burden of GBS.

Within Australia there is considerable variation in clinical practice in both the prevention of GBS sepsis in neonates and in practitioner opinions as to the appropriate approach to screening for and treatment of GBS [10]. Such variation in views amongst obstetricians and neonatologists reflects uncertainty about the application of differing hospital guidelines.

The current strategy at The Women's and Children's Hospital (WCH) in Adelaide for the prevention of GBS infection in the newborn includes the administration of prophylactic antibiotics during labour to women identified as being colonised with GBS, following universal screening with prenatal low vaginal cultures at 32 weeks gestation.

This study was designed to provide a scientific basis for optimum timing and method of GBS screening as specified in guidelines for antenatal care, to determine whether screening for GBS infection at 35–37 weeks gestation has better predictive values for colonisation at birth than screening at 31–33 weeks, to examine the test characteristics of a risk factor strategy and to determine the test characteristics of low vaginal swabs alone compared with a combination of perianal plus low vaginal swabs per colonisation during labour.

## Methods

### Study population

Women were eligible for inclusion if they had a singleton pregnancy, attended the Women's and Children's Hospital for their antenatal care over a 13-month period from May 1998 to May, 1999 and expected to deliver at that hospital at term. Women with previous GBS disease were included as were women enrolled in a shared care program between general practitioners and the hospital. Ethics committee approval was obtained from the Women's and Children's Hospital.

### Recruitment

Information sessions were held for antenatal clinic and labour ward staff prior to the commencement of recruitment and during the recruitment period, to familiarise staff with the study and incorporate their suggestions into the study protocol if appropriate. Women were informed about the study after their 18-week morphological scan and approached for consent at approximately 28 weeks gestation. Women who consented received vaginal and perianal swabs at 31–33 weeks, 35–38 weeks and during labour. A sample of these women participated in focus groups to explore their views about the collection of swabs antenatally and intra-partum, their attitudes to the prophylactic administration of antibiotics during labour and their understanding about GBS infection [11].

Although we had intended at the outset to recruit private patients, we could not develop cost-effective strategies for their involvement.

### Patient management

Participants had a low vaginal swab taken at 31–33 weeks for detection of GBS, the current protocol at the WCH. Standard recommendations for taking swabs were given to clinic staff to ensure that low vaginal swabs were taken without a speculum, by inserting the swab 2–3 cm. into the vagina and rotating the swab with a circular motion, leaving it in the vagina for approximately 5 seconds. A separate perianal swab was taken by gently rotating the swab around the anal margin for approximately five seconds. The swabs were placed in Stuart transport medium for transport to the laboratory within two hours.

At 35–37 weeks additional swabs (vaginal and perianal) were taken in the antenatal clinic by study staff following the protocol described above. The results of both screening swabs were made available to the caregivers. Women found on antenatal screening to be GBS carriers were recommended to have intrapartum antibiotics as per the hospital clinical guidelines.

When women were admitted in labour, a further vaginal and perianal swab was taken for culture by the admitting midwife to determine intrapartum colonisation. Note was made of whether the membranes had ruptured prior to the swab. Medical records for study participants contained an eye-catching sticker to remind labour ward staff that both a perianal and a vaginal swab needed to be taken. In addition, participants were given a card identifying them as participants in the "SWABS" study, to be handed to labour ward staff as a prompt for the taking of swabs. Study midwives provided inservice training on the protocol for taking labour swabs to staff in emergency, labour ward and the birthing centre.

### Microbiology

Upon receipt in the microbiology laboratory, the swabs were cultured on layered horse blood agar and inoculated into a selective broth (Todd Hewitt broth containing gentamicin 4 mg/L and nalidixic acid 15 mg/L). The agar plate was incubated at 35°C in a carbon dioxide-enriched environment for 18–24 hours, and the broth was incubated at 35°C overnight, subcultured onto layered horse blood agar and incubated as above. Plates were inspected for β-hemolytic colonies and Streptococci were identified according to standard laboratory procedures. Presumptive GBS colonies were confirmed using the Phadebact latex agglutination method. Growth on the plate was semiquantified as described by [12]. Growth from broth only was described as "scanty".

### Data collection and management

Data were collected to define the characteristics of the population including age, insurance status, socioeconomic status, parity, weight at booking, smoking status at booking, previous known GBS infection, asymptomatic bacteriuria, GBS screening at booking, previous preterm birth or preterm prelabour rupture of membranes. Women were classified as being at high risk if they had any of the following risk factors; GBS bacteruria at booking, birth at <37 weeks' gestation, prelabour rupture of membranes or temperature during labour of greater than or equal to 38 degrees Centigrade. Data were keyed into the study computer database (Microsoft ACCESS) with range checking, logic checking and verification of key fields.

### Analyses

Sensitivities, specificities, positive predictive values and negative predictive values for colonisation at birth, are reported with exact confidence intervals for antenatal screening at 31–33 (referred to as 32 weeks) and 35–37 weeks gestation (referred to as 36 weeks) for vaginal cultures alone (LVS), perianal cultures alone (PAS) and combined low vaginal and perianal cultures (either). Likelihood ratios were calculated to express the odds that a given level of a diagnostic test result would be expected in a woman colonised at term. Diagnostic odds ratios and their exact 95% confidence interval are an indication of the strength of the association between having a positive likelihood ratio and being diseased.

These test characteristics were compared to identify the best method of screening as determined by site of swab, timing and the interaction between site and timing, using a multinominal logit model with the weighted least squares method of estimation. The interaction term was not statistically significant and thus the results of the main effects models alone are reported [13].

Different screening strategies were examined to determine the relative value of screening at 8 weeks prior to expected birth (with the potential for lower predictive values but with the potential also for the identification of women colonised with GBS who may give birth between 32 and 35–37 weeks and screening closer to term or using risk factors to identify likely to be colonised at birth.

### Sample Size

The sample size was calculated assuming a weighted least squares analysis [14]. Calculations were based on assumptions concerning estimates of prevalence of GBS infection at the WCH at the time of routine screening (13%) and at birth (10%) (McDonald, personal communication). We assumed that the positivity rate at a 36-week screen would be 13%. Research published prior to the design of this study reported that a late antenatal screen had a sensitivity of approximately 87% [15]. Using a more conservative estimate of 80.5 percent, a sample size of approximately 839 was adequate to detect a sensitivity of 87% or greater. Alpha was set at 0.05 and β was selected to be 0.2. The correlation between estimated sensitivities at the two time periods was estimated to be approximately 0.7.

## Results

**Figure 1:**
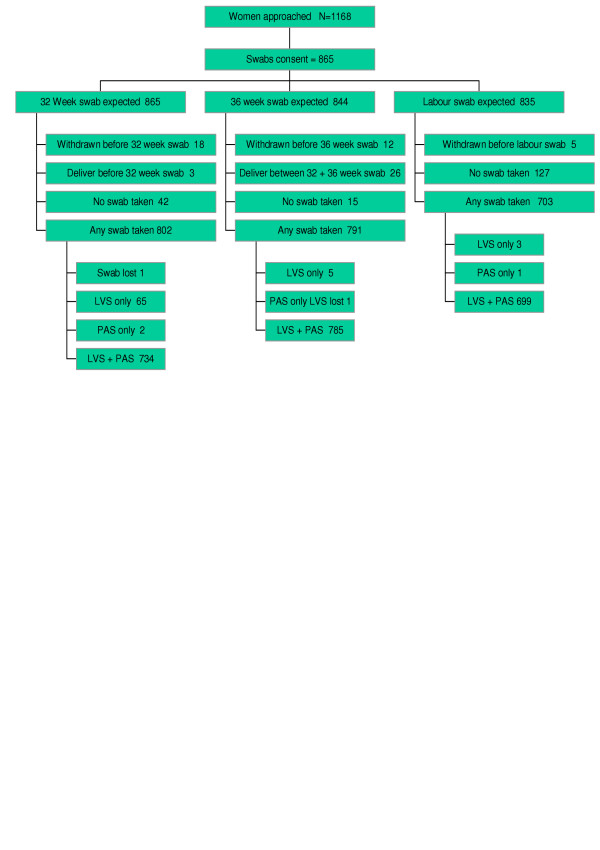
Recruitment and data collection. LVS Low vaginal swab PAS Perianal swab

A total of 865 women, of the 1168 approached, consented to participate in the SWABS study giving a participation rate of 74% (Figure 1). A number of these women (35) withdrew from the study over the period of follow-up, reflecting mobility of the patient population (9), reluctance of the woman or her partner to continue (21), a reaction to a positive test (3) and unknown reasons (2). Swabs were obtained from 93% of participants at 32 weeks, the time of the routine hospital antenatal screen for GBS. At 36 weeks swabs were taken from 94% of women who had neither given birth nor withdrawn while labour swabs were taken for 84% of women who had not withdrawn.

Study participants reflected the composition of the low-risk antental clinic at the Women's and Children's Hospital (Table 1) in their age, gravidity, parity and model of care. Thirty women (3.5%) were positive for GBS with bacteriuria at booking, although these results were not known for over 13% of participants. Of the 48% of women with a previous pregnancy who reached 20 weeks or more, 55 (13%) had a history of GBS carriage in a previous pregnancy. Only one woman reported a history of having a child with neonatal GBS sepsis.

**Table 1:** Characteristics of the 865 participants (Means and standard deviations or numbers and (percentages)).

Characteristic	Mean (sd) or Number (%)
Age Mean (sd)	28.0 (5.5)
Age group	
≤ 20	74 (8.6)
21–34	680 (78.6)
≥ 35	111 (12.8)
	
Parity	
0	447 (51.7)
1–3	402 (46.5)
≥ 4	16 (1.8)
	
Model of care (booking)	
Traditional + midwifery antenatal care	461 (53.3)
Birth Centre	185 (21.4)
GP Shared Care	219 (25.3)
	
Smoking at booking	185 (21.4%)
	
GBS bacteriuria at booking	
Positive (4% of all with known values)	30 (3.5%)
Unknown	117 (13.5%)
	
History of GBS among women with parity 1+ (n = 418)	
Positive in previous pregnancy (27.3% of known)	55 (13.2)
Unknown	184 (44%)
	
History of neonatal GBS sepsis n = 418	1 (0.2)
	
Unknown	26 (36.2)
History of	
Preterm birth	32 (7.7)
Preterm prelabour rupture of membranes	12 (2.9)
	
Sociodemographic characteristics	
Race	
Caucasian	818 (94.6)
Aboriginal/Torres Strait Islander	8 (0.9)
Asian	35 (4.0)
Other	4 (0.5)
	
Education	
School student	5 (0.6)
Left school aged < 16	55 (6.4)
Left school aged ≥ 16	369 (42.7)
Trade qualification	36 (4.2)
Certificate or Diploma	241 (27.9)
Bachelors Degree or higher	159 (18.4)

Colonisation rates were constant across the gestational ages examined, with approximately 20% of all participants colonised regardless of the gestational age of screening and the swab site. At all times, the colonisation rate was slightly higher with the perianal than the vaginal swab. (Table 2). Colonisation rates would have been reported as being between 14–17% if results for selective broth were not included, approximately 5% lower than those actually identified using the broth.

**Table 2:** Colonisation rates (%) and 95% confidence intervals by gestational age and swab sitea. Using selective broth

a. Using selective broth						
**Swab site**	**32 weeks gestation**		**36 weeks gestation**		**Labour**	
	**Prevalence**	**95 % CI**	**Prevalence**	**95% CI**	**Prevalence**	**95% CI**

**Low Vaginal Swab (LVS)**	19	16–21	20	17–23	21	18–24
**Perianal Swab (PAS)**	21	18–24	22	19–25	22	19–25
**Either LVS or PAS**	22	19–25	24	21–26	24	21–27

b. Removing "scanty" levels of colonisation as would occur in the absence of selective broth						

**Swab site**	**32 weeks gestation**		**36 weeks gestation**		**Labour**	
	**Prevalence**	**95 % CI**	**Prevalence**	**95% CI**	**Prevalence**	**95% CI**

**Low Vaginal Swab (LVS)**	14	11–16	15	13–18	17	14–20
**Perianal Swab (PAS)**	15	12–18	15	12–17	16	13–19
**Either LVS or PAS**	17	15–20	18	16–21	20	17–23

Using the risk factor algorithm a total of 146 women (18% of the 830 women who did not withdraw, 95% CI 15% – 20%) would have been identified as being eligible for intrapartum antibiotic chemoprophylaxis.

Analysis of the test characteristics associated with different screening schedules was undertaken for the subset of 600 (69%) study participants for whom complete data were available from testing at 32 weeks and 36 weeks gestation as well as in labour (Table 3). Analysis of the use of a risk factor protocol was undertaken for the 699 participants for whom a labour swab was taken (Table 3). It was apparent that tests at 36 weeks were more sensitive and had higher negative predictive values and lower Likelihood Ratio negative values than tests at 32 weeks. There were no statistically significant differences in specificity, positive predictive value or Likelihood Ratio positive values associated with the timing of screening. Having a positive perianal or low vaginal swab – the more inclusive definition, was a more sensitive test than low vaginal swab alone but with a trade-off in terms of specificity and positive predictive value. There was no statistical difference in the Likelihood Ratio positive, but Likelihood Ratio negative was lower using the more inclusive definition of positivity (Table 4). Classifying women as being at high risk was not sensitive (Table 3) and was not informative in predicting GBS status during labour (Likelihood ratio tests were not different than 1).

**Table 3:** Sensitivity, specificity, positive predictive value (PPV), negative predictive value (NPV), positive and negative likelihood ratios with 95% confidence intervals for screening at 32 weeks or 36 weeks, using low vaginal (LVS) and/or perianal (PAS) swabs for 600 women and for using a risk factor strategy*.

	**32 Weeks (n = 600)**						**36 Weeks (n = 600)**						(n= 699)	
	
	**LVS**		**PAS**		**Either**		**LVS**		**PAS**		**Either**			
	
	**Estimate + 95% CI**		**Estimate + 95% CI**		**Estimate + 95% CI**		**Estimate + 95% CI**		**Estimate + 95% CI**		**Estimate + 95% CI**		**Estimate + 95% CI**	
Sensitivity	63	54–71	70	61–77	72	63–79	73	65–81	76	67–83	81	73–87	19	13–26
**Specificity**	94	92–96	94	91–96	93	90–95	95	93–97	94	91–96	93	90–95	83	79–86
PPV*	77	68–84	76	68–84	75	66–82	82	74–88	78	70–85	77	69–84	25	18–34
**NPV**	90	87–92	91	89–94	92	89–94	92	90–95	93	90–95	94	92–96	77	73–80
**Likelihood ratio (+ve)**	11.3	7.6–16.7	11.2	7.7–16.2	10.1	7.2–14.3	15.5	10.2-23.6	12.1	8.4-17.5	11.7	8.3-16.6	1.10	0.76-1.59
**Likelihood ratio (-ve)**	0.39	0.31–0.49	0.32	0.25–0.42	0.30	0.23–0.40	0.28	0.21–0.37	0.26	0.19–0.35	0.21	0.15–0.29	0.98	0.90–1.06
**Diagnostic Odds Ratio**	28.7	16.4–50.6	34.5	19.8–60.4	33.4	19.4–57.9	55.4	30.1–103	46.5	26.1–83.1	56.7	31.4–103	1.12	0.69–1.79

* Risk factor strategy included women with GBS bacteruria at booking, birth at <37 weeks' gestation, prelabour rupture of membranes or temperature during labour of greater than or equal to 38 degrees Centrigrade.

**Table 4:** Analysis of timing and site of swabs.

	Timing (36 v 32 weeks)			Site (Either Lower Vaginal or Perianal vs Lower Vaginal Swab alone)		
	Difference (36 – 32)	95% Confidence Interval	p-value	Difference (Either vs LVS)	95% Confidence Interval	p-value

Sensitivity	8.8%	(1.2%, 16.3%)	0.023*	8.2%	(4.6%, 11.7%)	<0.001†
Specificity	0.6%	(-1.2%, 2.5%)	0.493	-1.8%	(-2.6%, -0.9%)	<0.001§/P >
Negative Predictive Value	2.3%	(0.3%, 4.3%)	0.026*	2.0%	(1.0%, 3.0%)	<0.001†
Positive Predictive Value	3.6%	(-2.8%, 9.9%)	0.269	-3.1%	(-5.8%, -0.5%)	0.019§/P >
Likelihood Ratio +	1.62	(-2.69, 5.92)	0.462	-1.59	(-3.68, 0.49)	0.134
Likelihood Ratio -	-0.09	(-0.18, -0.01)	0.021*	-0.08	(-0.12, -0.04)	<0.001†
Diagnostic Odds Ratio	14.3	(-8.06, 36.8)	0.210	3.63	(-4.99, 12.2)	0.409

* favours swabs taken at 36 weeks over 32 weeks

† favours either site over LVS only

§favours LVS only over either site

Multivariate analysis examined the impact of timing (screening at 36 rather than 32 weeks) and method of screening (low vaginal swabs or both low vaginal and perianal swabs) (Table 4). The interaction between method and timing was not significant. It was clear that there is an unequivocal benefit associated with delaying screening until 36 weeks. The results for method of screening were less definitive. Although sensitivity, negative predictive value and likelihood ratio negative were improved using a combined low vaginal and perianal swabbing regimen, the LVS swab alone was associated with higher specificity and positive predictive value.

## Discussion

This study has demonstrated that colonisation during pregnancy with Group B Streptococcus is common amongst a low risk antenatal population. Regardless of the timing of the testing, approximately 20% of women were identified with a positive swab and therefore would have been eligible, using the hospital protocol, for antibiotic use during labour. Although this colonisation rate is a little lower than that reported by Yancy et al. in their investigation of timing of swabs in 826 women (26.5% vs approx 20% in this study) there were slight differences in the study population with the former study excluding women who had received antibiotics within a week prior to birth. The test characteristics reported from the Yancy study (Sensitivity 87%, Specificity 96%, PPV 87%, NPV 96%) were all stronger than in this current study (36 weeks screen: Sensitivity 81%, Specificity 93%, PPV 77%, NPV 94%). Likelihood ratios and diagnostic odds ratios were not reported for the former study.

This study provides clear evidence about screening timing and strategy in order to identify women colonised with GBS in labour, with more equivocal evidence about methods. Screening for GBS infection at 35–37 weeks gestation has better test characteristics and predictive values for colonisation at birth than screening at 31–33 weeks. As the hospital in which this research was undertaken has a policy of routine administration of antibiotics to women at risk of preterm birth, a delay in the timing of screening would not exclude those women at higher risk for GBS infection. In an environment in which this was not policy however, screening at 35–37 weeks may miss a particularly high-risk group.

We have defined 'high risk' as GBS bacteruria at booking, preterm birth <37 weeks' gestation, prelabour rupture of membranes or pyrexia in labour (temperature greater than or equal to 38 degrees Centrigrade). The test characteristics of a screening strategy using these risk factors are relatively poor although the use of a non-independent reference standard (colonisation in labour identified following LVS and PVS) is an issue. This finding reinforces those from a multisite study sponsored by the Centers for Disease Control [16] whose guidelines state that a risk-based strategy is not supported.

### Low vaginal swabs, perinanal, or combined?

The use of both low vaginal swabs and perianal swabs identifies a higher proportion of colonised women. Whether in fact this higher antepartum detection rate will contribute to lower rates of neonatal infection and morbidity has yet to be determined. The additional costs of such an approach would need to be examined to determine whether hospital guidelines should be altered.

There is variability in screening practices in clinical practice nationally and worldwide. The companion paper from this study reporting the results of qualitative interviews with participants highlights that pregnant women are keen to do everything possible to ensure that they have a healthy liveborn infant and, that swabbing is not seen as particularly intrusive. Although these women expressed little concern about the potential adverse effects of antibiotic use [11] such concern is an appropriate one for healthcare workers.

## Conclusion

This study has many strengths. A very high proportion of eligible patients agreed to participate and follow-up rates were high. The approach used to taking swabs and to their subsequent analysis is in accord with current best practice. A more detailed approach to the statistical analysis of the data is presented than has been reported in prior research. The study had the ability to examine the potential impact of a high risk strategy and a screening strategy among the same women. What this study cannot do is provide much needed direct evidence about the relative effectiveness of different strategies in terms of prevention of Early Onset GBS. Such evidence would require the conduct of very large randomised controlled trials which to date have not been seen to be feasible. For this reason, evidence from studies such as this is essential for institutions developing screening policies.

## List of abbreviations used

CI Confidence Interval

EOGBS Early Onset Group B Streptococcal Sepsis

GBS Group B Streptococcus

LVS Low vaginal swabs

NPV Negative predictive value

PAS Perianal swabs

PPV Positive predictive value

WCH Women's and Children's Hospital

## Declaration of competing interests

The author(s) declare that they have no competing interests.

## Authors' contributions

JH contributed to conception and design, coordination of data collection, analysis and interpretation of data, drafting and revising the article and approval for publication.

HMcD contributed to the design, data collection, analysis and interpretation of microbiological data and participated in drafting and revising the article

PD contributed to the study design, interpretation of results and revision of the article

CC contributed to the conception and design, data collection, interpretation of results and drafting and revision of the article.

## Pre-publication history

The pre-publication history for this paper can be accessed here:



## References

[B1] Schuchat A (1998). Epidemiology of Group B Streptococcal Disease in the United States: Shifting Paradigms. Clin Microbiol Rev.

[B2] Isaacs D, Royle JA (1999). Intrapartum antibiotics and early onset neonatal sepsis caused by group B streptococcus and by other organisms in Australia. Australasian Study Group for Neonatal Infections. Pediatr Infect Dis J.

[B3] Heath PT, Balfour G, Weisner AM, Efstratiou A, Lamagni TL, Tighe H, O'Connell LAF, Cafferkey M, Verlander NO, Nicoll A, McCartney AC, on behalf of the PHLS GBS Working Group (2004). Group B streptococcal disease in UK and Irish infants younger than 90 days. Lancet.

[B4] Boyer KM, Gadzala CA, Burd LI, Fisher DE, Paton JB, Gotoff SP (1983). Selective intrapartum chemoprophylaxis of neonatal group B streptococcal early-onset disease. I. Epidemiologic rationale. J Infect Dis.

[B5] McDonald HM, O'Loughlin JA, Jolley P, Vigneswaran R, McDonald PJ (1992). Prenatal microbiological risk factors associated with preterm birth. Br J Obstet Gynaecol.

[B6] Gilbert GL, Hewitt MC, Turner CM, Leeder SR (2002). Epidemiology and predictive values of risk factors for neonatal group B streptococcal sepsis. Aust N Z J Obstet Gynaecol.

[B7] Benitz WE, Gould JB, Druzin ML (1999). Risk factors for early-onset Group B Streptococcal sepsis: estimation of odds ratios by critical literature review. Pediatrics.

[B8] Centers for Disease Control and Prevention (2002). Prevention of perinatal group B streptococcal disease. MMWR.

[B9] Royal College of Obstetricians and Gynaecologists (2003). Prevention of Early Onset Neonatal Group B Streptococcal Disease. RCOG Guideline.

[B10] McLaughlin K, Crowther C (2000). Universal antenatal group B streptococcus screening? The opinions of obstetricians and neonatologists within Australia. Aust NZ J Obstet Gynaecol.

[B11] Darbyshire P, Collins C, McDonald HM, Hiller JE (2003). Taking antenatal GBS seriously: women's experiences of screening and perceptions of risk. Birth.

[B12] Rotheram EB, Schick NF (1969). Nonclostidial anaerobic bacteria in septic abortion. Am J Med.

[B13] Grizzle JE, Sturmer CF, Koch GG (1969). Analysis of categorical data by linear models. Biometrics.

[B14] Rochon J (1989). The application of the GSK method to the determination of minimum sample sizes. Biometrics.

[B15] Yancey MK, Schuchat A, Brown LK, Ventura VL, Markenson GR (1996). The accuracy of late antenatal screening cultures in predicting genital Group B Streptococcal colonization at delivery. Obstet Gynecol.

[B16] Schrag SJ, Zell ER, Lynfield R, Roome A, Arnold KE, Craig AS, Harrison LH, Reingold A, Stefonek K, Smith G, Gamble M, Schuchat A (2002). A population-based comparison of strategies to prevent early-onset group B streptococcal disease in neonates. N Engl J Med.

